# Chromatin fiber structural motifs as regulatory hubs of genome function?

**DOI:** 10.1042/EBC20180065

**Published:** 2019-04-09

**Authors:** Manuela Moraru, Thomas Schalch

**Affiliations:** Leicester Institute for Structural and Chemical Biology, Department of Molecular and Cell Biology, University of Leicester, Leicester LE1 9HN, U.K.

**Keywords:** cryo-electron microscopy, chromatin, crystallography, small-angle scattering, transcription

## Abstract

Nucleosomes cover eukaryotic genomes like beads on a string and play a central role in regulating genome function. Isolated strings of nucleosomes have the potential to compact and form higher order chromatin structures, such as the well-characterized 30-nm fiber. However, despite tremendous advances in observing chromatin fibers *in situ* it has not been possible to confirm that regularly ordered fibers represent a prevalent structural level in the folding of chromosomes. Instead, it appears that folding at a larger scale than the nucleosome involves a variety of random structures with fractal characteristics. Nevertheless, recent progress provides evidence for the existence of structural motifs in chromatin fibers, potentially localized to strategic sites in the genome. Here we review the current understanding of chromatin fiber folding and the emerging roles that oligonucleosomal motifs play in the regulation of genome function.

## Introduction

The physical form of eukaryotic genomes is a complex of DNA, RNA, and proteins referred to as ‘chromatin’. Chromatin compacts the long and fragile DNA molecules into the confines of the cell’s nucleus and protects them. At the same time it facilitates and regulates access to the underlying DNA sequence for vital processes such as transcription, replication, DNA repair, and chromosome segregation. It is therefore not surprising that chromatin is deeply involved in adaptation to environmental changes, in development, ageing, and in disease etiology [1,2].

The basic building block of chromatin is the nucleosome, which is constituted by an octameric histone core wrapped by 145–147 bp of DNA in approximately 1.7 helical turns [3,4]. The histone core is assembled from two heterodimers of histones H2A and H2B, which pack symmetrically onto the central tetramer formed by histones H3 and H4 [5]. The linker histone H1 associates with nucleosomes and binds to the short stretches of linker DNA that connect nucleosomes as they cover the entire genome to form the chromatin fiber [6]. The structure and function of the chromatin fiber will be the focus of this review.

The primary role of histones is to neutralize the DNA charge and together with inorganic and small organic ions they facilitate the compaction of the genome [7]. In contrast to small ions, histones guide the DNA into a tight superhelical path and thereby form the nucleosome, a uniquely compact and highly regular structural unit of chromatin. In addition to the 145–147 bp of core DNA, nucleosomes occupy a variable length of linker DNA, and the combined length of core and linker DNA is referred to as nucleosome repeat length (NRL), which varies between 150 and 250 bp depending on cell type and organism [8]. Along the entire length of the genome the nature of chromatin changes constantly, correlating with gene activity, for example as observed for active euchromatic or silent heterochromatic regions. These changes are evident in the density and localization of nucleosomes, in specific post-translational modifications on histones and DNA, and in the deposition of histone variants such as for example the centromere-specific cenH3/CENP-A, which replaces H3 at the centromeres [9–11].

**Figure 1:**
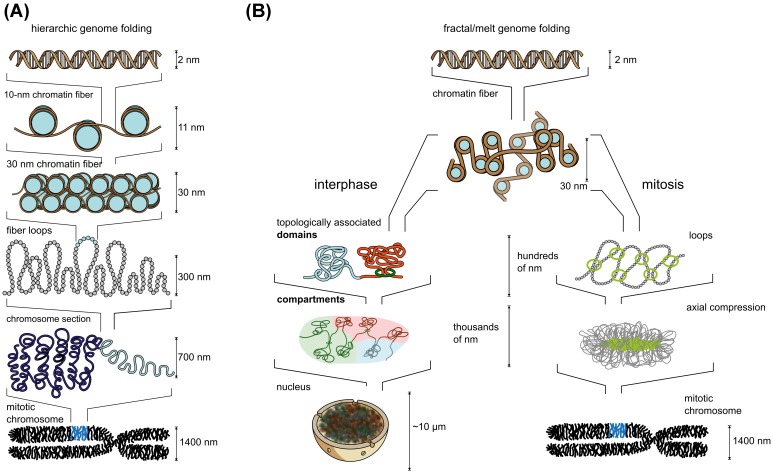
Classical versus state-of-the art chromatin model (**A**) The hierarchical model of chromatin folding [18] in comparison with (**B**) the dynamic and fractal chromatin folding model, which is emerging from recent developments.

Recent advances have shown that at the kilo- to megabase scale, the chromatin fiber organizes into preferentially interacting regions, referred to as Chromosomally Interacting Domains (CIDs) or Topologically Associating Domains (TADs), which for example regulate enhancer–promoter interactions and replication units through the action of cohesin and CTCF [12,13]. TADs further associate with other domains of similar properties into compartments of heterochromatin and euchromatin [14,15]. At the chromosomal level, chromosomes occupy non-overlapping territories in interphase, and compact to their highest degree as metaphase chromosomes during mitosis [16,17]. Classical models postulate that the chromatin fiber assumes a compacted ‘30-nm fiber’ structure in heterochromatin and the metaphase chromosome, while existing as a relaxed ‘11-nm fiber’ (the nucleosome diameter) in euchromatin (Figure 1A) [18]. As will be discussed further below, this classical model of chromatin folding has failed to hold up in face of the technological progress made in recent years and a much more random organization has emerged (Figure 1B). In particular the structure and functional role of the chromatin fiber remains a mystery despite great advances in the understanding of the nucleosome and of long-range genome regulation by TADs. We propose here that oligonucleosomal motifs play an important role at short genomic distances. In the coming sections, we will assemble the currently available evidence of chromatin fiber motifs and their role in local genome function.

## Architectural principles of isolated chromatin fibers

For the last 50 years, the structure of the chromatin fiber has been analyzed intensively by biochemical approaches, by light and electron microscopy (EM), by small angle diffraction and X-ray crystallography.

**Figure 2:**
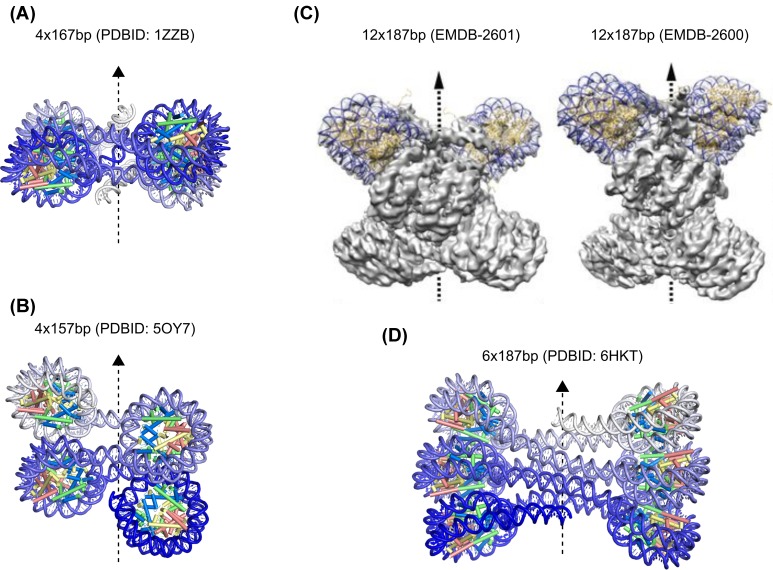
X-ray and cryo-EM structures of chromatin fiber motifs (**A**) Crystal structure of the 167-bp NRL tetranucleosome [28]. (**B**) Crystal structure of the 157-bp NRL tetranucleosome [30]. (**C**) Cryo-EM structure of 187 and 177 bp NRL dodecanucleosomes [29]. Adapted from ref. [29] with permission from AAAS. (**D**) Crystal structure of the 187-bp NRL hexanucleosome [27].

First groundbreaking work using EM on chromatin isolated from cells revealed the existence of filaments with a beads-on-a-string appearance [19] and further investigation revealed that mono- and divalent salts as well as H1 drive the structural transition from 11- to 30-nm fibers [20,21]. For a long time, the internal structure of the chromatin fiber was contentious due to a lack of resolution with most models being based on the one-start solenoid model [20] or the two-start zigzag models [22–24]. Significant progress has been made on the question of chromatin fiber structure *in vitro* by cross-linking approaches [25–27] and by X-ray and cryo-EM structures with sufficient resolution to resolve the linker DNA [27–30] (Figure 2). Both biochemistry and high-resolution structures support a two-start model for compact chromatin fibers with regularly spaced nucleosomes. While a consensus has emerged for two-start structures of fibers with short NRL of 150–180 bp, some controversy remains for fibers with long NRLs (>190 bp). Based on fiber diameters observed in cryo-EM of long fibers and based on complex modeling of single molecule force spectroscopy data an interdigitated solenoid model has been proposed [31–34]. This model is incompatible with the high-resolution cryo-EM and cross-linking data for chromatin fibers reconstituted with H1 and long linker lengths, which support the two-start model [25,27,29].

While the two-start architecture is emerging as the most prevalent fold of the chromatin fiber it needs to be emphasized that the flexibility of the DNA string and the weak interactions between nucleosomes permit a great amount of structural heterogeneity that includes solenoid-type nearest neighbor as well as long-range interactions [26,35]. Single-molecule FRET experiments further highlight the highly dynamic nature of chromatin fibers in solution [36,37].

High-resolution methods such as X-ray crystallography and cryo-EM as well as most biophysical methods rely on structurally homogeneous chromatin fibers with strongly positioned nucleosomes and uniform linker lengths. However, natural chromatin is far more complex, containing variation in nucleosome positions, linker length, and histone content. Therefore, a key question is how regular or irregular are natural chromatin fibers?

## Structure of the chromatin fiber in the nucleus

Bulk measurements of NRL using micrococcal nuclease have been performed on many species, revealing that NRLs of natural chromatin span a range between 150 and 250 bp. Yeast species have short NRLs of 150–170 bp, while many animal cells assume values of approximately 180–200 bp [8]. Notable exceptions are the cerebral cortex neurons with 160–170 bp repeat length [38,39]. Furthermore, telomeric chromatin has been observed to have particularly short NRLs of 157 bp [40,41]. Further, NRLs strongly depend on H1 levels, lending support to the hypothesis that H1 binds and neutralizes the linker DNA and thereby prevents encroachment of core histones [42–45].

When the relative frequencies of NRLs are compared, a 10-bp (one helical turn of DNA) quantization becomes apparent [46]. Also within individual genomes a relatively high degree of quantization is evident in DNase I ladders of internucleosomal fragments, which show a preferential linker length of {10n + 5} bp with n = 0, 1, 2, 3, 4, … for *Saccharomyces cerevisiae* and chicken erythrocytes [47,48]. Chemical cleavage mapping of nucleosome positions has further demonstrated the {10n + 5} bp quantization in *S. cerevisiae, S. pombe* and also in mouse embryonic stem cells (mESCs), although on top of a stronger background signal, which indicates increased heterogeneity in linker lengths in the mESCs [49–51]. Comparison of fibers with 10n bp quantized linkers versus 10n + 5 bp linkers suggests that {10n + 5} bp chromatin fibers compact less efficiently and that they are associated with transcriptionally active genomic regions [51–53].

The genomics area has seen a large increase in nucleosome mapping studies that combine micrococcal nuclease digestion or chemical cleavage with next-generation sequencing (NGS) [54,55]. These experiments provide detailed information on nucleosome positions across genomes and have revealed that nucleosome-free regions around transcription start sites are flanked by regularly spaced nucleosomes. Regularity in other parts of the genome like for example heterochromatin has not been evident in these analyses. Recent single-cell approaches in mouse and *Drosophila melanogaster* have made significant advances in the question of nucleosome spacing on genomes and show that heterochromatic regions and non-transcribed genes are more regularly spaced than transcriptionally active regions [45,56]. These data suggest that silent regions of the genome are uniformly spaced, but that they lack phasing in contrast with active genes, where the nucleosome-free regions provide a barrier from which neighboring nucleosomes are spaced in regular intervals. Strong phasing is also observed at certain transcription factor-binding sites, in particular at CTCF sites, which demarcate TAD boundaries and insulator elements [51,57,58].

**Figure 3:**
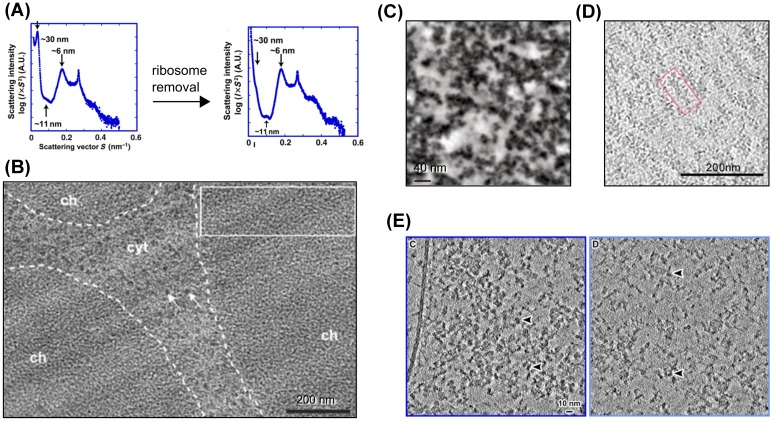
Chromatin observed *in vivo* (**A**) Small-angle X-ray scattering of HeLa mitotic chromosomes shows loss of the 30-nm peak upon ribosomes removal [62]. Reproduced from ref. [62] with permission from EMBO. (**B**) Micrograph of an approximately 50-nm thick frozen hydrated section of a mitotic HeLa S3 cell that contains three regions of chromosomes (ch) separated by cytoplasm (cyt) [63]. White arrows indicate ribosomes. Reproduced from ref. [63], Copyright (2008), National Academy of Sciences, U.S.A. (**C**) Tomographic section of a mitotic chromosome in U2OS cells obtained by ChromEMT [64]. Reprinted from ref. [64] with permission from AAAS. (**D**) A 10-nm-thick slice through a 3D reconstruction of a cryosection from a chicken erythrocyte nucleus [65]. A longitudinally sectioned 30-nm fiber is indicated by the red box. Reprinted from ref. [65]. (**E**) Tomographic slice (10 nm) of heterochromatin (left) and euchromatin (right) from cryosections in HeLa cells [66]. Individual nucleosomes are indicated by arrowheads. Reproduced from ref. [66] under CC BY-NC-SA 3.0 license.

Given the primary distribution of nucleosomes on the DNA sequence, how do the strings of nucleosomes fold into secondary and tertiary structures? Seminal experiments using small angle X-ray scattering on nuclei revealed the presence of 40-30 nm periodicities, which were assigned to chromatin fibers [23,59]. However, more recent scattering experiments have not confirmed these results and have instead shown that ribosomal contaminations can give rise to 30-nm peaks in the spectrum [60,61] (Figure 3A).

Traditional *in situ* EM that relies on cross-linking, staining, and resin embedding has a deleterious impact on the fine structure of chromatin, and therefore structural analyses of chromatin fibers have been unreliable until the establishment of cryo-EM technology, which enabled imaging of frozen hydrated sections of cells in vitreous ice [67,68] (Figure 3B, D, E). Application of the *in situ* cryo-EM technologies to chromatin has made it clear that there is enormous complexity in the chromatin fibers structure, which depends on organism, cell type, cell cycle, and genome context. On one hand, there are cells with clear evidence for 30-nm fibers such as sperm from marine species like sea urchin or sea stars and from chicken erythrocytes [65,69,70] (Figure 3D). However, most other eukaryotic cells including yeasts, picoplankton, and mammalian cells investigated by cryo-EM do not show evidence for regular 30-nm fibers [62,63,66,67,70–75] (Figure 3). All these experiments observe a grainy texture for chromatin containing more or less dense regions with characteristic periodicities of 6 and 11 nm. These observations have led to the proposal of an interdigitated phase of chromatin, where chromatin fibers ‘melt’ into each other through close contacts between non-neighbor nucleosomes [63]. Furthermore, rheological experiments with labeled tracer molecules in the nucleus, as well as X-ray and neutron scattering data support the formation of a highly random chromatin organization with fractal characteristics in the entire range of nuclear organization above the nucleosome level [60,61,76,77]. In contrast, a recent DNA proximal staining method has been developed to visualize chromatin in the nucleus at high resolution (ChromEMT) [64], which shows discrete strings of chromatin with diameters between 5 and 24 nm. With all likelihood, this apparent contradiction between a melt and fibrous state is caused by cross-linking, dehydration, and embedding procedures used in ChromEMT, which distort the ultrastructure of chromatin fibers (compare panels B and C in Figure 3).

We conclude that, except for some terminally differentiated and transcriptionally inert cell types, native chromatin fibers in the nuclear environment appear as interdigitated and highly irregular structures.

## Structural motifs of the chromatin fiber

The observed global irregularity of chromatin fibers is likely to obscure well-defined oligonucleosomal structures that might be associated with specific genomic features and that potentially play a role in genome function. In the last section of this review, we will provide an overview of the evidence that supports the existence of functionally important oligonucleosomal motifs.

An innovative approach to studying chromatin organization in living cells was pioneered by Rydberg et al. [78], using synchrotron radiation to generate spatially correlated DNA breaks. The resulting patterns support the existence of a two-start fiber architecture in the nucleus. Exploitation of the same approach in combination with NGS and computer modeling showed that different regions of the genome assume different cleavage patterns, pointing to the existence of non-random features depending on the genomic context [79]. Single molecule STORM imaging of intact nuclei has furthermore revealed, that nucleosomes cluster together in “clutches”, which are potentially correlating with defined oligonucleosomal structures [80].

Chromosome conformation capture experiments with nucleosomal resolution (Micro-C) have recently been developed to study chromatin fiber structure *in vivo* [81,82]. These experiments do not show evidence for long-range order in the chromatin fiber, but are consistent with tri- and tetranucleosomal units as chromatin fiber folding motifs. The micro-C data have further provided support that tetranucleosomes are linked with the function of the FACT histone chaperone complex in gene transcription [83]. Chromatin-associated complexes like FACT often contain multiple chromatin binding and histone modification-specific reader domains [84,85]. It is therefore highly likely that the stereochemistry of the chromatin fiber is involved in recruiting chromatin-associated cellular processes.

**Figure 4:**
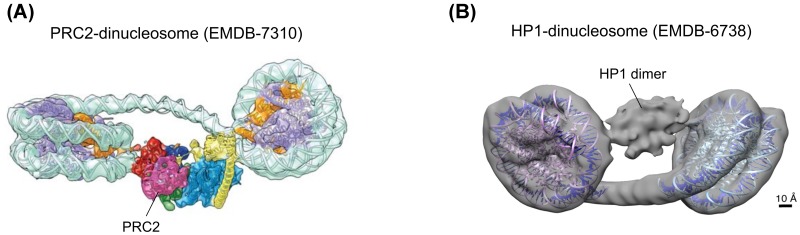
Dinucleosomes as substrates of chromatin effector complexes (**A**) Cryo-EM structure of PRC2 bound to a dinucleosome [88]. Adapted from ref [88], Copyright (2018), with permission from Springer Nature. (**B**) HP1 bound to a dinucleosome [91]. Adapted from ref [91], Copyright (2018), with permission from Elsevier.

Dinucleosomes are the simplest oligonucleosomal substrates, but as all other chromatin fiber motifs they are nevertheless structurally quite diverse. This is due to DNA linker variation, which not only changes the distance between the two nucleosomes, but also has a huge impact on relative orientation, since addition of every base pair induces a change in relative rotation between the two nucleosomes of 36°. There are multiple lines of evidence that dinucleosomes are preferred substrates for chromatin-modifying complexes. For example the polycomb complex PRC2 prefers di- and oligonucleosomes as substrates over mononucleosomes [86,87], and the recent cryo-EM structure of PRC2 in complex with a dinucleosome provides a detailed snapshot of how PRC2 uses an existing H3K27me3 mark on one nucleosome to methylate H3K27 on the neighboring nucleosome [88] (Figure 4A). Interestingly, the structural work also shows that PRC2 is able to adapt to dinucleosomes with opposite rotational orientation. A similar mechanism might be at work in the histone deacetylase complex Rpd3S, which binds dinucleosomes with higher affinity as it recognizes the H3K36me mark to deacetylate acetyl-lysine residues [89]. Furthermore, nucleosome remodelers like ISWI have been proposed to recognize a dinucleosomal substrate in order to modify DNA linker length and evenly space nucleosomes [90].

The heterochromatin protein HP1 is a dimer with two H3K9me3 binding chromodomains, which has the potential to engage with two nucleosomes. Recently, HP1 bound to a dinucleosome has been visualized by cryo-EM [91] (Figure 4B). Nevertheless, HP1 binding is highly dynamic [92,93], and it is therefore very likely that it bridges nucleosomes *in cis* as well as *in trans* (between fibers) to condense and segregate heterochromatin.

Trinucleosomes form V-shaped motifs that compact upon increasing salt levels [94], and trinucleosomes have furthermore been readily observed *in situ* by cryo-EM [66] and are therefore likely to serve as functional motifs. For example, the yeast silencing complex consisting of Sir2, Sir3, and Sir4 has been shown to bind trinucleosomes. The Sir2-3-4 complex binds DNA through Sir4 and the non-acetylated histone H4 tails through the BAH domain of Sir3, with Sir3 binding being sensitive to H3K79 methylation [95,96]. Particularly in combination with the NAD-derived deacetylation product of Sir2, O-AADPR, the oligomeric Sir2–3–4 complex shows preference for trinucleosomal units [97].

Tetranucleosomes have shown to be crystallizable biochemical entities at NRLs of 157 and 167 bp [28,30] (Figure 2A,B). They have further been observed to form structural units of longer chromatin fibers by cryo-EM [29] (Figure 2C). It is therefore likely that tetranucleosomes play a role as biologically relevant units of the chromatin fiber. The repeat lengths in all those structural studies correspond to the 10n linker class, and the fact that many other NRL constructs between 150 and 177 bp have failed to yield well diffracting crystals suggests that the 10n class forms the structurally most homogeneous oligonucleosomes [30]. *S. pombe* has a short NRL with an average of 152 bp, and the tetranucleosome crystal structures with 157 bp NRL reveal a relatively open structure with minimal face-to-face contact in the chromatin fiber [30]. Interestingly, *in situ* cryo-EM of *S. pombe* chromatin is consistent with the crystal structures since face-to-face nucleosome contacts were not observed [74]. In contrast, stacking arrangements of nucleosomes were observed in human cell lines [75]. The NRL of the 167 bp tetranucleosome structure is relatively close to the *S. cerevisiae* average NRL of 165 bp and shows a compact, dumbbell-shaped arrangement of two pairs of nucleosomes facing each other [28]. Comparison of the crystal structure to cryo-electron tomography of frozen hydrated yeast cells failed to detect regularly occurring similar structures [73]. Instead, small oligonucleosomal clusters assuming various configurations have been observed in the generally dense, but randomly packed *S. cerevisiae* chromatin. Tetranucleosomes have further been implicated in chromatin compaction through the PRC1 complex. *In vitro*, PRC1 binds and compacts preferentially tetranucleosomes [98]. However, it remains to be determined if this applies to the physiological situation in the nucleus.

Description of penta- and longer oligonucleosomes as preferred substrates of chromatin-associated complexes is rare and longer regular structures have not been detected in mammalian cells [66]. One documented example of a complex associated with longer chromatin fragments is the DNA methyltrasferase DNMT3B, whose chromatin binding increases with higher levels of H1 and with longer, more compact chromatin fiber substrates *in vitro* and in cells [99].

## Conclusions

While the nucleosome is a structurally well-defined entity, the chain of nucleosomes appears to assume highly variable architectures. *In vitro* there is clear evidence for the potential of the chromatin fiber to assume compact, regular structures. However, in the crowded environment of the nucleus these fibers appear to interdigitate and ‘melt’ into each other in a highly random fashion. At the same time, there is evidence supporting the existence of small oligonucleosomal structures in the nucleus as well as evidence supporting oligonucleosomes as the physiological substrates for large chromatin-associated complexes. Elucidating the role of oligonucleosomal structures in genome function therefore remains a key topic. The technologies most likely to provide breakthroughs are cryo-EM and micro-C derived NGS approaches. High-resolution cryo-EM combined with focused ion beam (FIB) milling to create undistorted, ultrathin sections of cells is already being employed [74] and will further be of great use to explore genome structure. The main fundamental challenge with genome imaging is the lack of sequence context for the observed structures. This challenge might be amenable to new, non-denaturing labeling technologies using CRISPR, Talen, or Zn-finger technologies to mark specific genomic features with heavy atom compounds. On the other hand, further development of micro-C technologies toward higher resolution is a promising avenue that will directly relate structure and genome features. The major challenge for micro-C will be to boost resolution and genome coverage. This might be tackled by the development of more specific cross-linking approaches to obtain more accurate structural constraints than with the commonly used formaldehyde. Further gain in resolution will also come from development of modeling algorithms that extract maximum information from the NGS data as very recently proposed [100]. We are looking forward to seeing how the powerful tools available today in combination with further developments will be opening new avenues to explore this long-standing question.

## Summary

- In contrast with regular structures of chromatin fibers observed *in vitro*, chromatin fibers *in situ* manifest a highly random organization.
- Oligonucleosomes serve as preferred substrates for chromatin-associated complexes.
- We propose that defined oligonucleosomal structures play an important role in chromatin biology.

## Abbreviations

EM: electron microscopy
mESC: mouse embryonic stem cell
NGS: next-generation sequencing
NRL: nucleosome repeat length
TAD: topologically associating domain
H3K27me3: histone H3 with trimethylated lysine 27
